# Supplementary material for: Molecular signatures of resilience to Alzheimer’s disease in neocortical layer 4 neurons
Source: Nat Commun. 2026 Jan 31;17:2223. doi: 10.1038/s41467-026-68920-4 (PMC12963381; doi:10.1038/s41467-026-68920-4)
Supplement: Supplementary file 1 — Supplementary Information [file 41467_2026_68920_MOESM1_ESM.pdf]

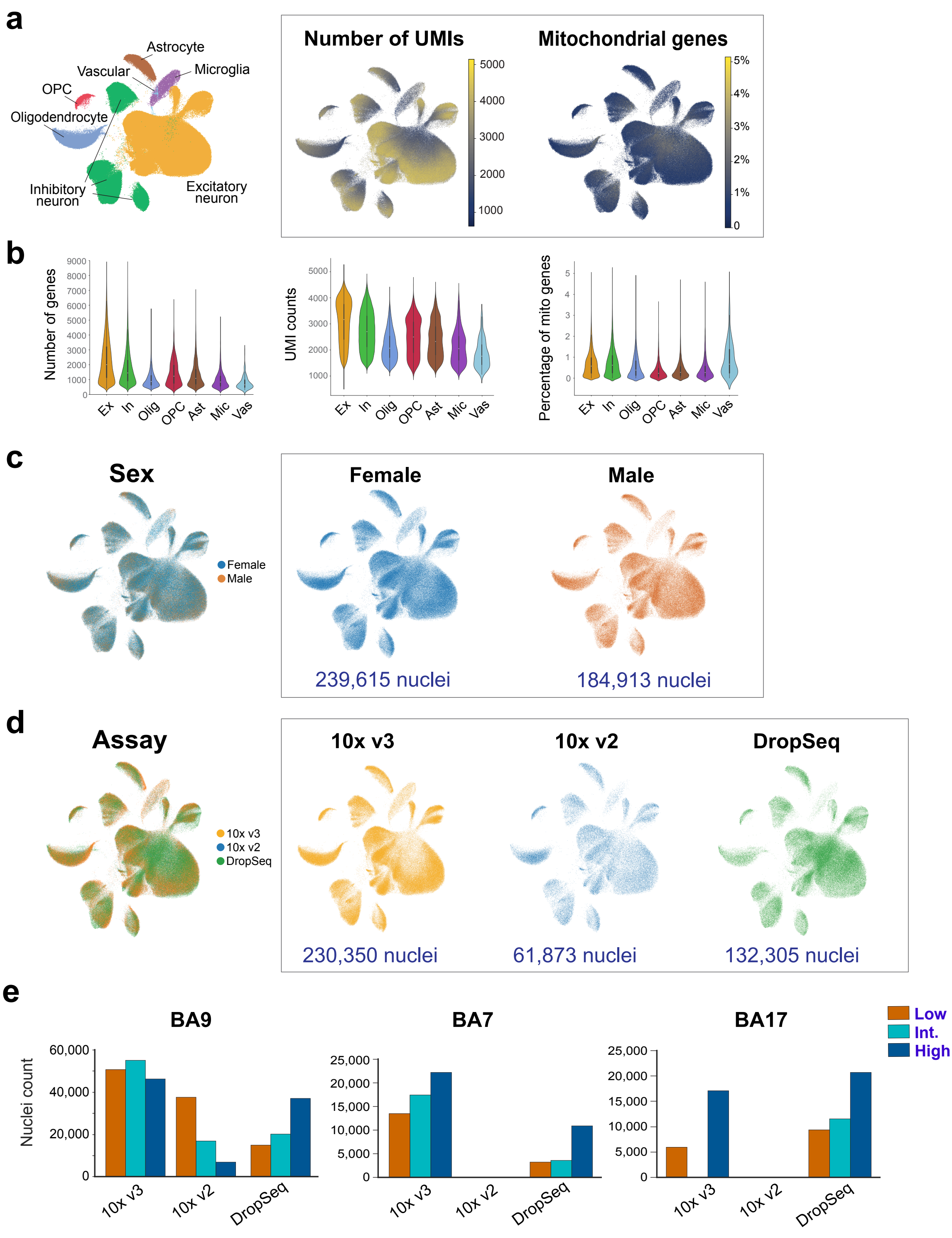

Supplementary Fig. 1. snRNA-seq quality control (QC)

a, UMAP plot of the annotated major cell types after the removal of low-quality nuclei and doublets (left) and feature plots displaying the median number of UMIs per nucleus and the proportion of transcripts mapping to mitochondrial genes (right). **b**, Violin plots showing the median number of genes, median number of UMIs, and percentage of mitochondrial genes within major cell types. **c**, UMAP plots splitting the datasets by sex. **d**, UMAP plots splitting the datasets by profiling assay (DropSeq, 10x v2, 10x v3). **e**, Bar plots representing the number of nuclei obtained from each profiling assay within each brain region (BA9, BA7, BA17) and disease stage group (low, intermediate, late) after QC. Source data are provided as a Source Data file.

a

Cluster-specific gene sets

Ex1:CUX2-SERPINE2-LAMP5	*SERPINE2, LAMP5, SEMA3C, LINC00507, GNAL, HPCAL1, THSD7A, MAML2*
Ex2:CUX2-RORB-GLIS3-LRRC2	*LRRC2, CNGB1, GRIK1, LINC00326, GRB14, PRKG2, LINC01478, LINC00470*
Ex3:CUX2-RORB-GLIS3-SV2C	*SV2C, GYG2, MFGE8, ENPEP, TEC, TXK, SYT2, GPAT3, ANKRD29, ONECUT2*
Ex4:CUX2-RORB-COL5A2-CLMN	*CLMN, CCDC168, PRSS12, TEX30, ADAMTS6, CCDC68, PAH, VIPR2, CALB1, RASSF3*
Ex5:RORB-CUX2-EYA4-LAMA3	*EYA4, TRPC3, LAMA3, VAV3, NXPH1, IL2RA, PCAT1, KCNIP1, KCNH8*
Ex6:RORB-MME	*MME, COL22A1, CCDC168, GNG4, TEX30, BTNL9, ITGA11, GPR26, PHLDB2, SYT17*
Ex7:RORB-GABRG1	*GABRG1, LYPD6B, ANXA1, ALDH1A1, LOXHD1, PTK7, GOLIM4, VIPR2, ST8SIA4, MERTK*
Ex8:RORB-TLL1-TMTC4	*TMTC4, KCNK2, MKX, PTPRZ1, ARHGAP29, ASPM, CLMP, SFTPD, GPR161*
Ex9:RORB-TLL1-PCP4-MID1	*MID1, PCP4, PLD1, BTBD11, GPRIN3, KCNK2, DAPK2*
Ex10:RORB-TLL1-PCP4-TYR	*TYR, CMTM8, MYBPC1, GRIN3A, NOX4, RNF182, TLL1, RBM20, ADTRP, CCDC68*
Ex11:RORB-ADGRL4	*ADGRL4, NPFFR2, MEGF10, PKD2L1, LYPD6B, ARHGAP15, SULF2, RUNX2, SMIM36, ITGA11*
Ex12:THEMIS-PRRX1	*PRRX1, PXDN, MCUB, DCHS2, DKK2, ARHGAP18, LINC00343*
Ex13:THEMIS-POSTN	*POSTN, SMYD1, OLFML2B, GAS2L3, SNTB1, MCTP2, NR4A2, PRLR, PDLIM5, KLHL1*
Ex14:FEZF2-HTR2C	*HTR2C, NXPH2, PDE9A, CARD11, KCNIP1, CD36, ERG, ITGA8, ROR1, ROBO3*
Ex15:FEZF2-ADRA1A	*ADRA1A, RREB1, CACNA1H, RNF152, SLC5A8, POU3F1, ANGPT1, LRP2, PDE9A, ERG*
Ex16:FEZF2-SYT6	*SYT6, SULF1, SH2D1B, SMIM36, SEMA5A, MCC, VAV2, ANKRD18A, ARHGAP6, CNTNAP3B*
Ex17:FEZF2-ZFHX3-SEMA3D	*SEMA3D, RASEF, NPFFR2, MCTP2, CDC14A, ITGA1, ITGA8, RNF220, RBM20, MDFIC*
Ex18:FEZF2-ZFHX3-SCUBE1	*SCUBE1, VCAN, CCN2, SLC15A5, FEZF2, SULF1, RNF220, VWA2, ERG, FAT4*

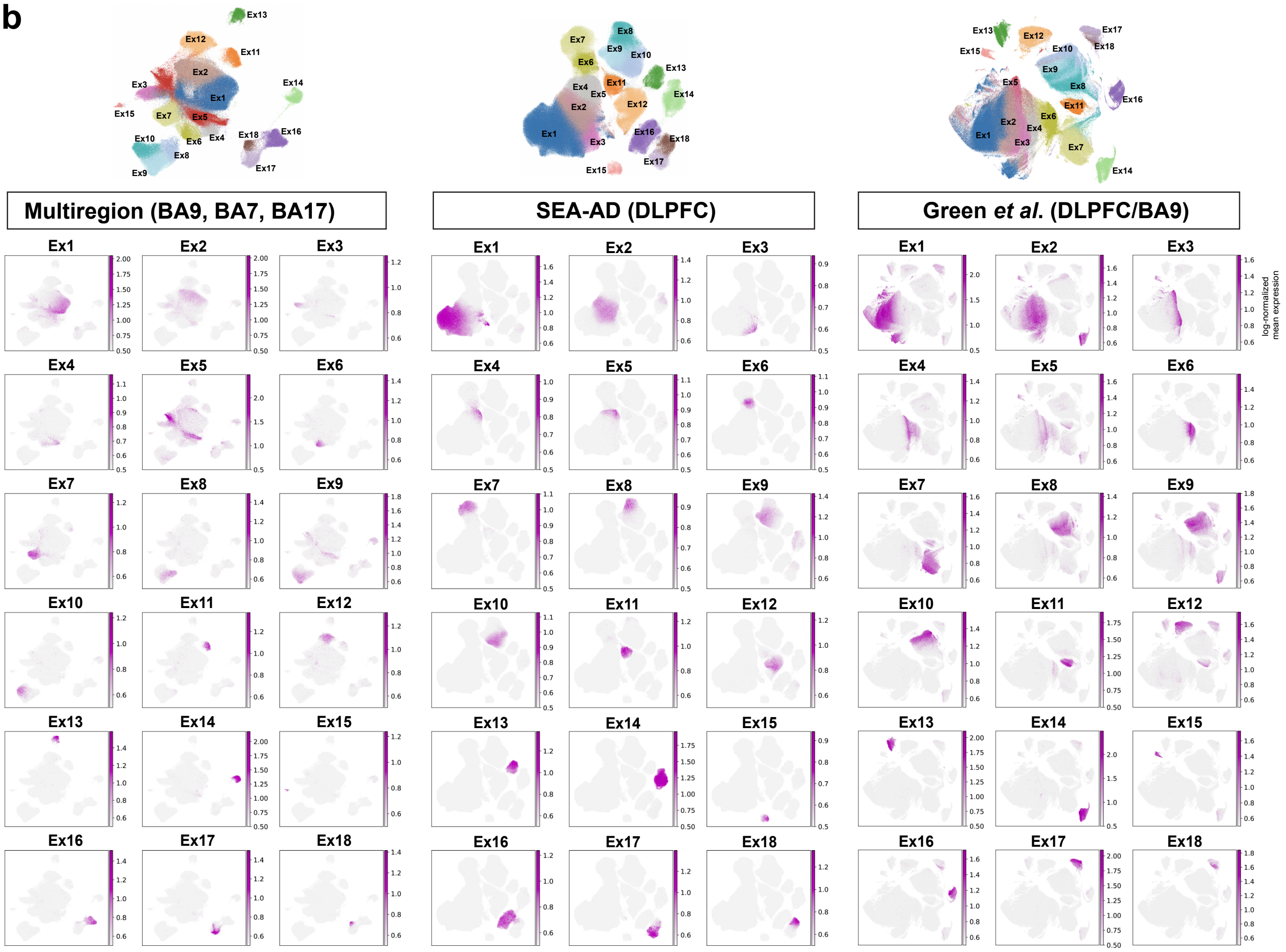

Supplementary Fig. 2. Identification of excitatory neuron clusters across datasets

a, Cluster-defining gene sets for each excitatory neuron cluster (Ex1-18). **b**, UMAP visualization of the excitatory neuron clusters (top) and gene expression UMAPs for the genes included in the cluster-specific gene sets (bottom), across three datasets: the present manuscript (multiregion; BA9, BA7, BA17), SEA-AD (DLPFC) (PMID: 39402379), and Green and colleagues (DLPFC/BA9) (PMID: 39198642). Clusters in the public datasets were predicted using scANVI.

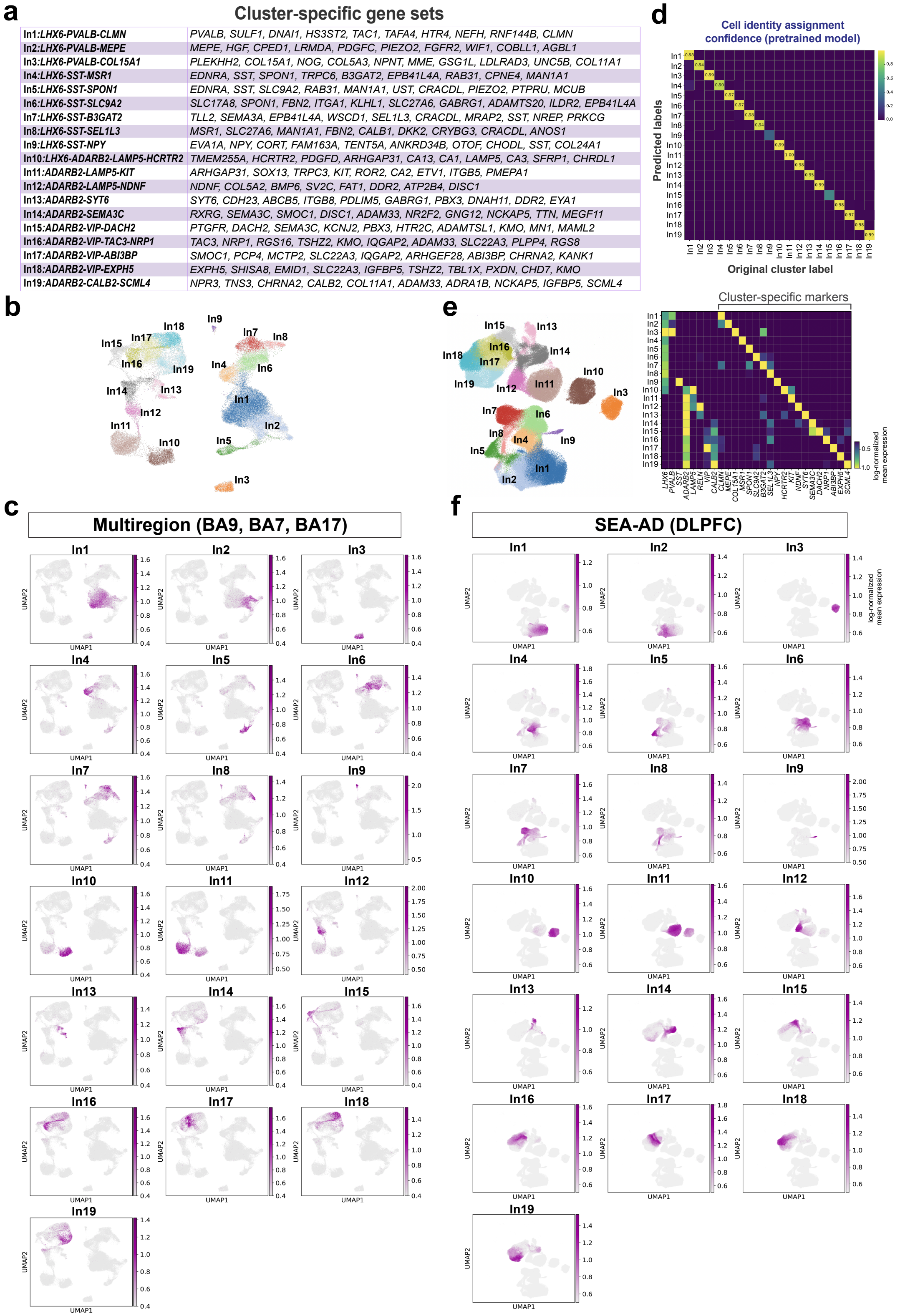

Cluster-specific gene sets

In1:*LHX6-PVALB-CLMN*	*PVALB, SULF1, DNAI1, HS3ST2, TAC1, TAFA4, HTR4, NEFH, RNF144B, CLMN*
In2:*LHX6-PVALB-MEPE*	*MEPE, HGF, CPED1, LRMDA, PDGFC, PIEZO2, FGFR2, WIF1, COBLL1, AGBL1*
In3:*LHX6-PVALB-COL15A1*	*PLEKHH2, COL15A1, NOG, COL5A3, NPNT, MME, GSG1L, LDLRAD3, UNC5B, COL11A1*
In4:*LHX6-SST-MSR1*	*EDNRA, SST, SPON1, TRPC6, B3GAT2, EPB41L4A, RAB31, CPNE4, MAN1A1*
In5:*LHX6-SST-SPON1*	*EDNRA, SST, SLC9A2, RAB31, MAN1A1, UST, CRACDL, PIEZO2, PTPRU, MCUB*
In6:*LHX6-SST-SLC9A2*	*SLC17A8, SPON1, FBN2, ITGA1, KLHL1, SLC27A6, GABRG1, ADAMTS20, ILDR2, EPB41L4A*
In7:*LHX6-SST-B3GAT2*	*TLL2, SEMA3A, EPB41L4A, WSCD1, SEL1L3, CRACDL, MRAP2, SST, NREP, PRKCG*
In8:*LHX6-SST-SEL1L3*	*MSR1, SLC27A6, MAN1A1, FBN2, CALB1, DKK2, CRYBG3, CRACDL, ANOS1*
In9:*LHX6-SST-NPY*	*EVA1A, NPY, CORT, FAM163A, TENT5A, ANKRD34B, OTOF, CHODL, SST, COL24A1*
In10:*LHX6-ADARB2-LAMP5-HCRTR2*	*TMEM255A, HCRTR2, PDGFD, ARHGAP31, CA13, CA1, LAMP5, CA3, SFRP1, CHRDL1*
In11:*ADARB2-LAMP5-KIT*	*ARHGAP31, SOX13, TRPC3, KIT, ROR2, CA2, ETV1, ITGB5, PMEPA1*
In12:*ADARB2-LAMP5-NDNF*	*NDNF, COL5A2, BMP6, SV2C, FAT1, DDR2, ATP2B4, DISC1*
In13:*ADARB2-SYT6*	*SYT6, CDH23, ABCB5, ITGB8, PDLIM5, GABRG1, PBX3, DNAH11, DDR2, EYA1*
In14:*ADARB2-SEMA3C*	*RXRG, SEMA3C, SMOC1, DISC1, ADAM33, NR2F2, GNG12, NCKAP5, TTN, MEGF11*
In15:*ADARB2-VIP-DACH2*	*PTGFR, DACH2, SEMA3C, KCNJ2, PBX3, HTR2C, ADAMTSL1, KMO, MN1, MAML2*
In16:*ADARB2-VIP-TAC3-NRP1*	*TAC3, NRP1, RGS16, TSHZ2, KMO, IQGAP2, ADAM33, SLC22A3, PLPP4, RGS8*
In17:*ADARB2-VIP-ABI3BP*	*SMOC1, PCP4, MCTP2, SLC22A3, IQGAP2, ARHGEF28, ABI3BP, CHRNA2, KANK1*
In18:*ADARB2-VIP-EXPH5*	*EXPH5, SHISA8, EMID1, SLC22A3, IGFBP5, TSHZ2, TBL1X, PXDN, CHD7, KMO*
In19:*ADARB2-CALB2-SCML4*	*NPR3, TNS3, CHRNA2, CALB2, COL11A1, ADAM33, ADRA1B, NCKAP5, IGFBP5, SCML4*

Supplementary Fig. 3. Identification of inhibitory neuron clusters across datasets

a, Cluster-defining gene sets for each inhibitory neuron cluster (In1-19). **b**, UMAP visualization of the inhibitory neuron clusters **c**, UMAP visualization of gene expression for the genes included in the cluster-defining gene sets. **d**, Heatmap showing the assignment confidence scores for each inhibitory cluster from the pretrained model using scANVI. **e**, UMAP and heatmap showing the predicted inhibitory clusters in a reference, publicly available dataset from the prefrontal cortex (SEA-AD DLPFC; PMID: 39402379) and their marker genes. **f**, UMAP visualization of gene expression for the cluster-defining gene sets in the SEA-AD (DLPFC) dataset. Clusters in the public dataset were predicted using scANVI.

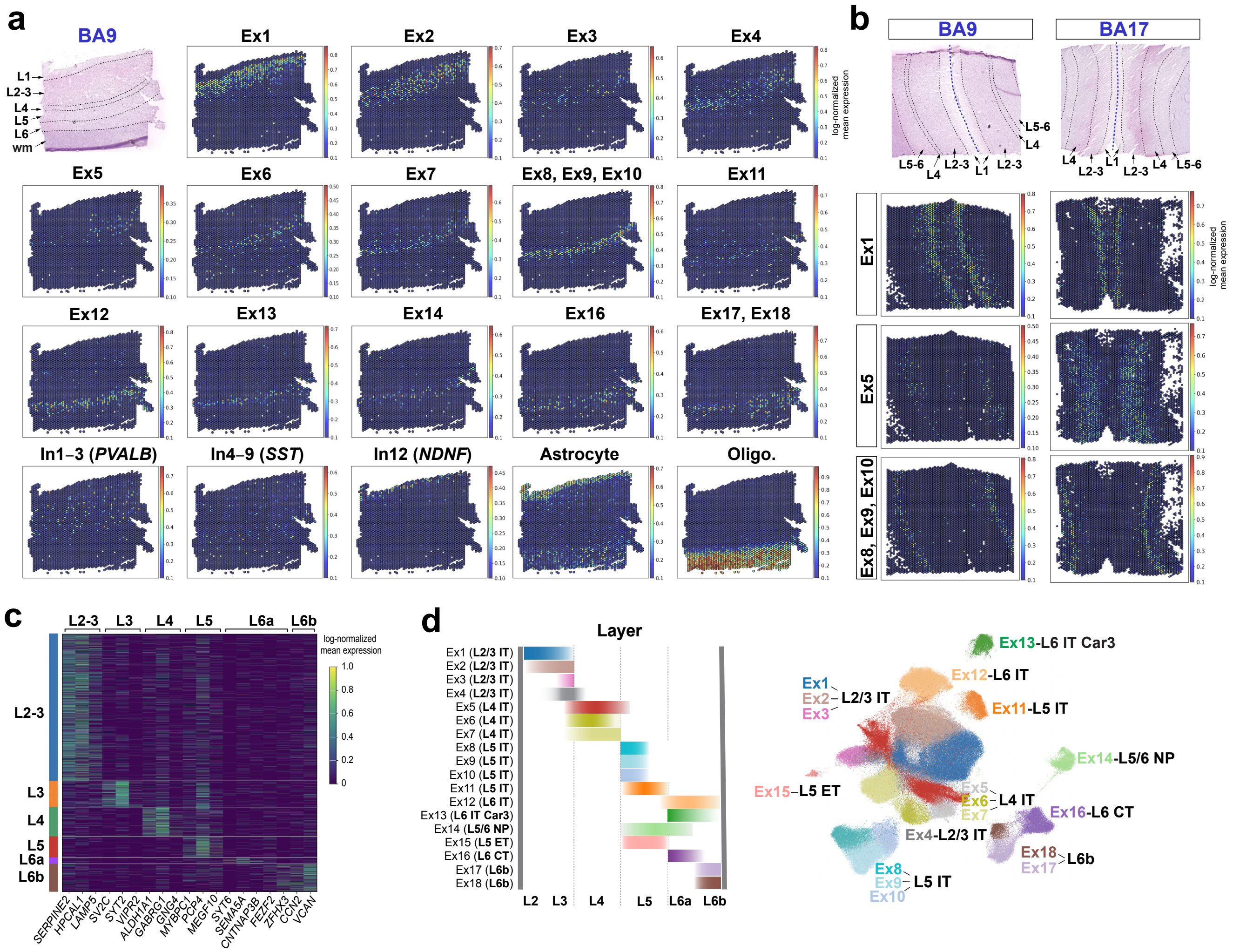

Supplementary Fig. 4. Layer-specific localization of excitatory neuronal subtypes in BA9 and BA17 by Visium

a, Spatial distribution of excitatory clusters in a representative BA9 section from a control donor. The H&E-stained tissue section indicates the boundaries between neocortical layers (top left). Expression of the excitatory subtypes annotated by snRNA-seq and visualized in the 10x Visium space using Stereoscope, highlighting the layer-restricted expression of each excitatory cluster. Smaller, overlapping clusters (Ex8/Ex9/Ex10 and Ex17/Ex18) are visualized together. *PVALB*⁺ interneurons span layers 2-5, *SST*⁺ interneurons span layers 1-6, whereas the *NDNF* interneuron subtype is restricted to L1. Astrocytes in L1 and in white matter and oligodendrocytes in white matter are shown for reference. **b**, Differences in L4 thickness and gene expression between BA9 and BA17. Representative H&E sections (top) and expression maps (bottom) illustrate the laminar expression of Ex5 (*RORB/CUX2/EYA4/LAMA3*) in L4, which is expanded in BA17, compared with Ex1 in superficial L2/3, and Ex8/Ex9/Ex10 in L5. **c**, Heatmap showing unsupervised layer distribution of top marker genes included in the 10x Visium gene panel. **d**, Summary of the 18 annotated excitatory subtypes and their layer-specific spatial distribution, incorporating annotations for excitatory neuron subclasses (L2/3 IT, L4 IT, L5 IT, L6 IT, L6 IT car3, L5/6 NP, L6b, and L6 CT) from the BICCN reference framework (PMID: 37824655).

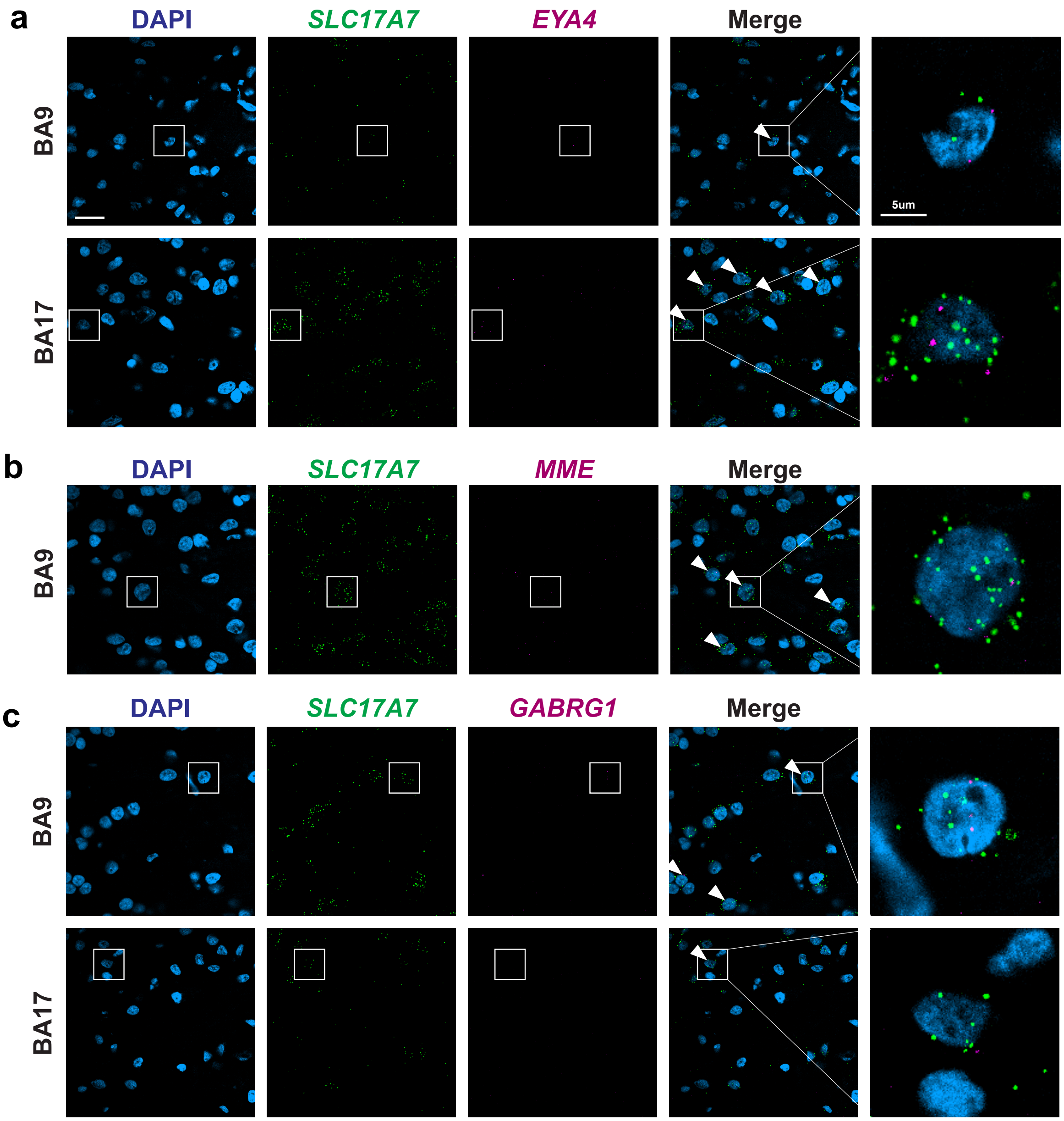

Supplementary Fig. 5. Expression of L4 excitatory neurons markers by RNAscope

a-c. Representative confocal images of layer 4 excitatory neurons from BA9 and BA17 healthy control tissue sections, co-stained with double fluorescent RNAscope ISH for *SLC17A7* (green signal) and either *EYA4* (**a**), *MME* (**b**), or *GABRG1* (**c**) (magenta signal), along with DAPI. *MME*-expressing excitatory neurons were not identified in BA17. Arrowheads indicate co-stained cells. Scale bar: 20 μm.

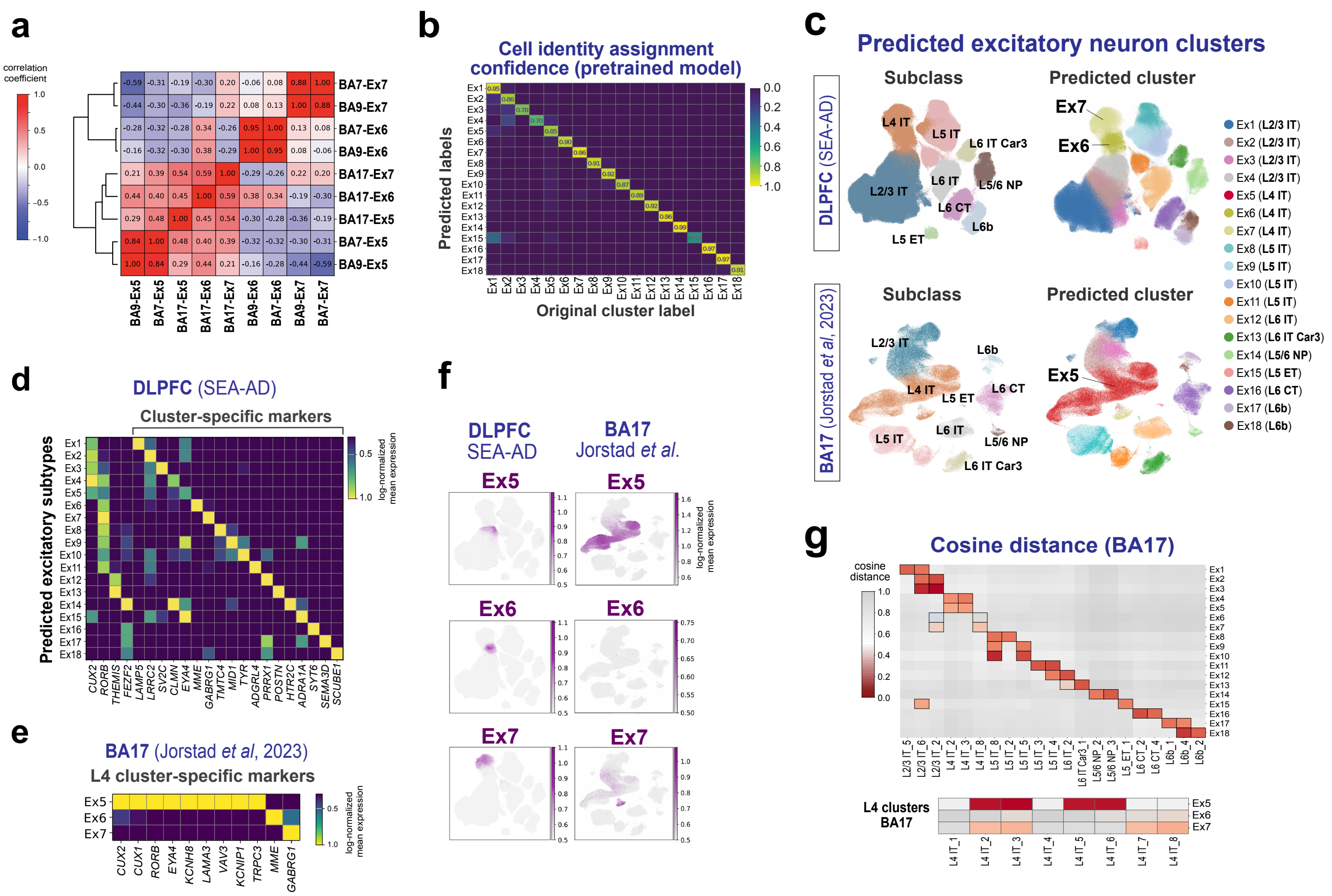

Supplementary Fig. 6. Prediction of annotated excitatory clusters in independent datasets using scANVI

a, Pearson correlation coefficients measuring similarity in gene expression between L4 clusters (Ex5, Ex6, and Ex7) across different brain regions (BA9, BA7, and BA17). **b**, Heatmap showing the assignment confidence scores for each excitatory cluster from the pretrained model using scANVI. **c**, UMAPs showing the author-annotated excitatory subclasses (left) and predicted excitatory clusters (right) in reference datasets from prefrontal cortex (SEA-AD DLPFC; PMID: 39402379) and primary visual cortex (PMID: 37824655). **d,e**, Gene expression heatmaps showing the normalized expression of cluster-specific marker genes in the predicted clusters from SEA-AD DLPFC (**d**) and BA17 L4 markers in the predicted clusters from subsetted L4 BA17 (**e**). **f**, Gene expression UMAPs of our cluster-defining gene sets for Ex5, Ex6, and Ex7 in the reference datasets from BA9 and BA17. **g**, Cosine distance matrix based on 3,000 highly variable genes from our BA17 dataset and the BA17 reference dataset demonstrating high similarity. In the top matrix, the three most closely related clusters, based on cosine distance, are depicted. In the bottom matrix, all L4 clusters are shown. Source data are provided as a Source Data file.

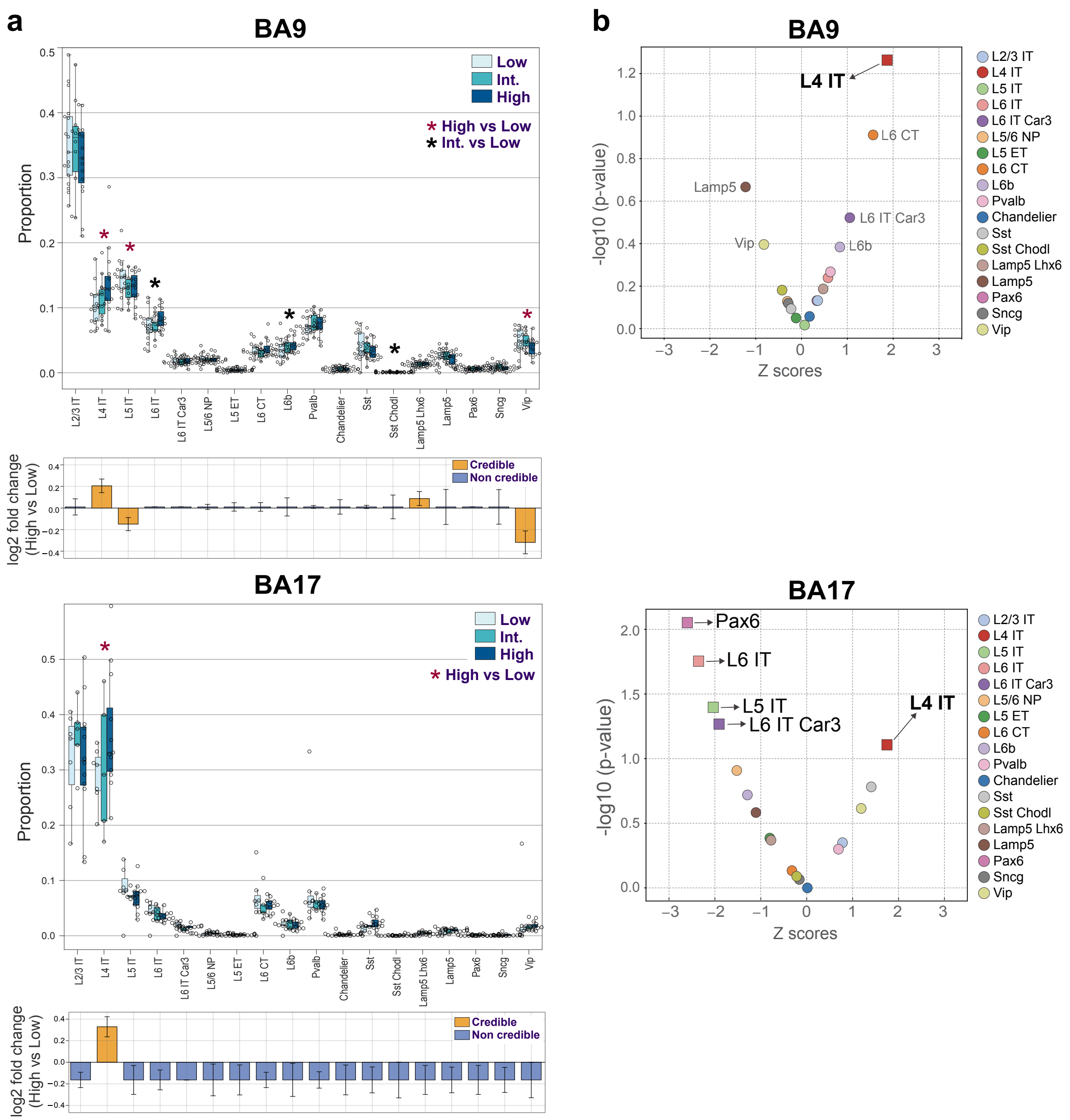

Supplementary Fig. 7. Relative preservation of L4 IT neurons in advanced AD

a, Box plots showing the relative abundance of neuronal populations, using reference annotations (BICCN) (PMID: 37824655). Neuronal cell compositions were estimated with scCODA. Individual donor proportions are overlaid as open circles. Data are presented as median (center line) and interquartile range (IQR; box limits); whiskers extend to the most extreme values within 1.5×IQR. Circles beyond the whiskers represent outliers. Sample sizes for BA9: low 17, intermediate 10, high 15 donors; BA17: low 7, intermediate 5, high 12 donors). Credible differences between high and low pathology groups (red asterisks) and between intermediate and low groups (black asterisks) are shown for clusters with a magnitude of change (log2-fold change) greater than 0.1, in either direction. Credible effects were defined at those with a posterior inclusion probability (PIP) > 0.95. The lower plots show the credible effects (highlighted in orange) along with the fold changes between high and low pathology groups; bars represent log2-fold change, and error bars indicate the standard error of the mean. **b**, Differential cell proportion analysis of neuronal populations between low and high disease groups using GLMM in BA9 and BA17. In BA9, L4 IT neurons showed an increased relative abundance in advanced AD, though this change did not reach statistical significance after correction for multiple comparisons (p-value = 0.03, FDR = 0.09). In BA17, L4 IT neurons showed a non-significant upward trend (p-value = 0.08), while Pax6 (p-value = 0.009), L6 IT (p = 0.018), L5 IT (p-value = 0.040), and L6 IT Car3 (p-value = 0.054) neuronal populations exhibited decreased relative abundance. These changes in BA17 were also not significant after correction for multiple comparisons. Source data are provided as a Source Data file.

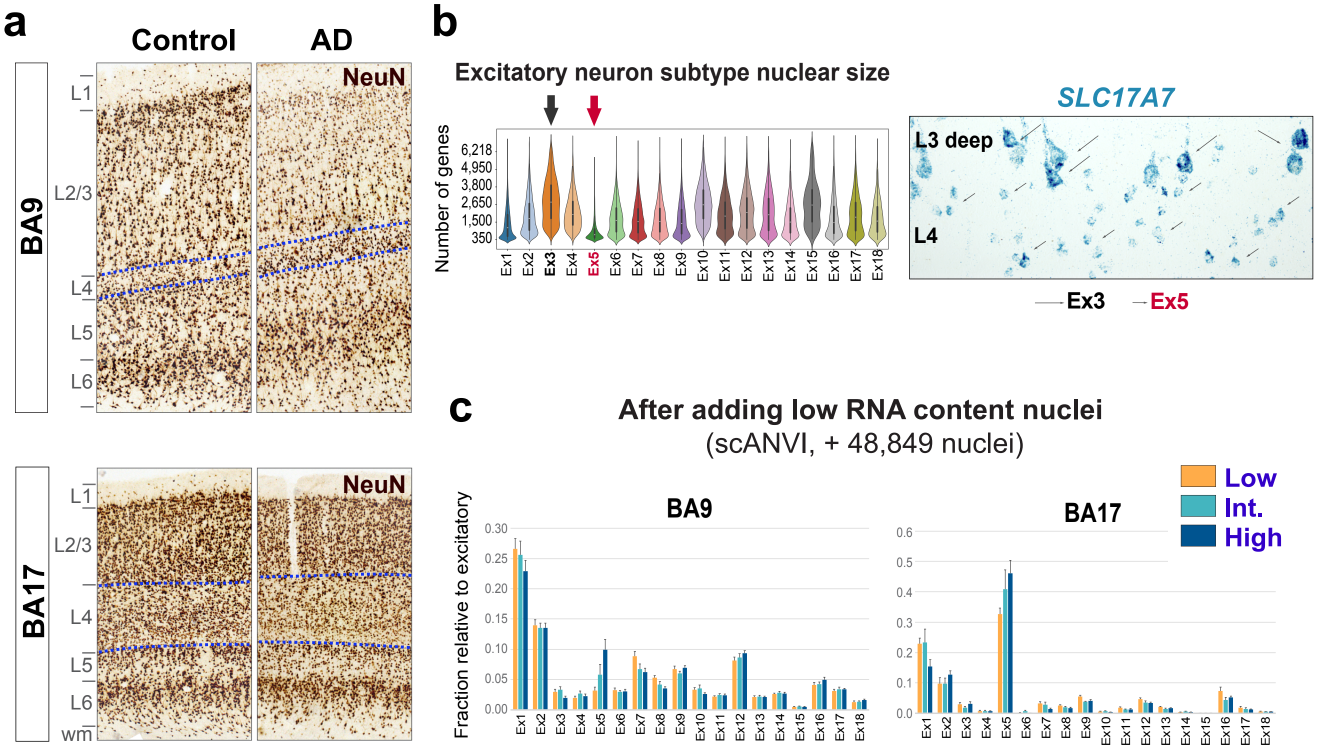

Supplementary Fig. 8. Assessment of neuronal composition and Ex5 cluster representation after QC filtering

a, NeuN immunostaining on 50-µm-thick sections through the neocortex highlights the loss of pyramidal neurons in L2/3 and the relative preservation of granule cells in L4 in Braak VI AD compared with controls in BA9. Dashed lines delineate L4. **b**, Violin plots representing the number of genes within each cluster serve as an indicator of their relative neuronal size. ISH for *SLC17A7* in a tissue section from BA9 illustrates the relatively small size of layer 4 neurons (Ex5; red arrowheads) and the large size of deep layer 3 neurons (Ex3; black arrows). **c**, Annotation of previously discarded nuclei due to their low gene content (between 200 and 300 genes per nucleus) using scANVI. The heatmap shows prediction confidence for each excitatory cluster (x-axis: reference cells; y-axis: query cells). The bar plots show the fractions of each excitatory subtype relative to all excitatory neurons in BA9 and BA17 for each disease group. After incorporating 48,849 additional nuclei into the dataset, the relative preservation of Ex5 remained. Source data are provided as a Source Data file.

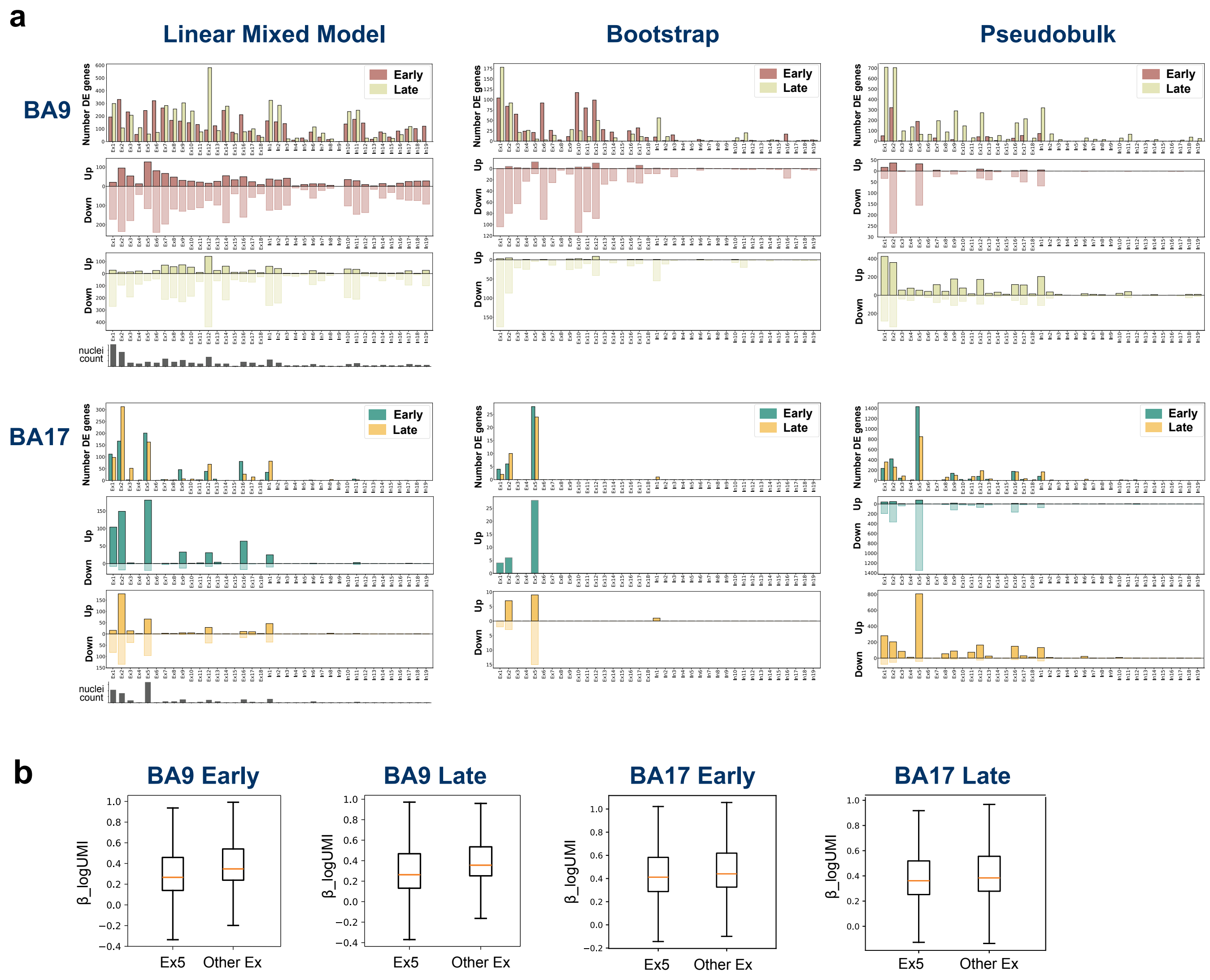

Supplementary Fig. 9. Number of DE genes identified using a linear mixed model, bootstrap, or pseudobulk

a, Bar plots illustrate the total numbers of DE genes, upregulated genes, and downregulated genes within each excitatory and inhibitory neuronal cluster at early and late disease stages in the BA9 and BA17. The number of nuclei per cluster is plotted for reference. Source data are provided as a Source Data file. **b**, Per-gene distributions of linear mixed model coefficients for sequencing depth (logUMI) in Ex5 neurons and other excitatory neuron types in BA9 and BA17, across early and late disease stages.

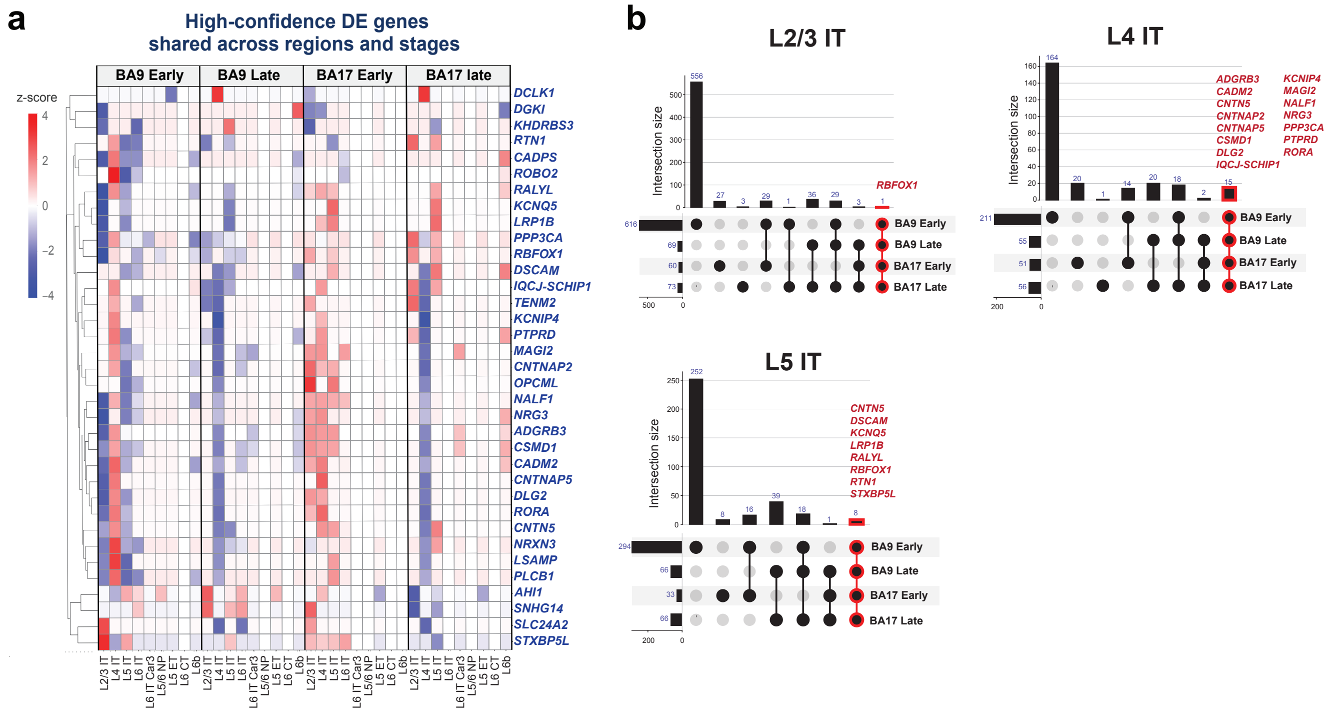

Supplementary Fig. 10. 'High-confidence' DE genes across reference layer-specific excitatory neuronal subtypes in AD neocortex

a,b, 'High-confidence' DE genes identified using a linear mixed model and either bootstrap, pseudobulk, or hdWGCNA analyses for each excitatory neuron subclass (L2/3 IT, L4 IT, L5 IT, L6 IT, L6 IT car3, L5/6 NP, L6b, and L6 CT). Nuclei with fewer than 500 genes were filtered out. 'Early' and 'late' DE genes correspond to comparisons between intermediate vs low and high vs intermediate AD pathology groups, respectively. Left: Gene expression heatmap of top 35 high-confidence DE genes shared across brain regions and disease stages. Colors indicate the average log-fold change obtained from the linear mixed model. Right: UpSet plots illustrating the intersection of high-confidence DE genes across BA9 and BA17 at early and late stages for L2/3 IT, L4 IT, L5 IT neurons. Rows correspond to each of the four conditions, and columns represent the intersections. Bar charts depict the numbers of intersecting genes. Genes highlighted in red are differentially expressed in all four conditions.

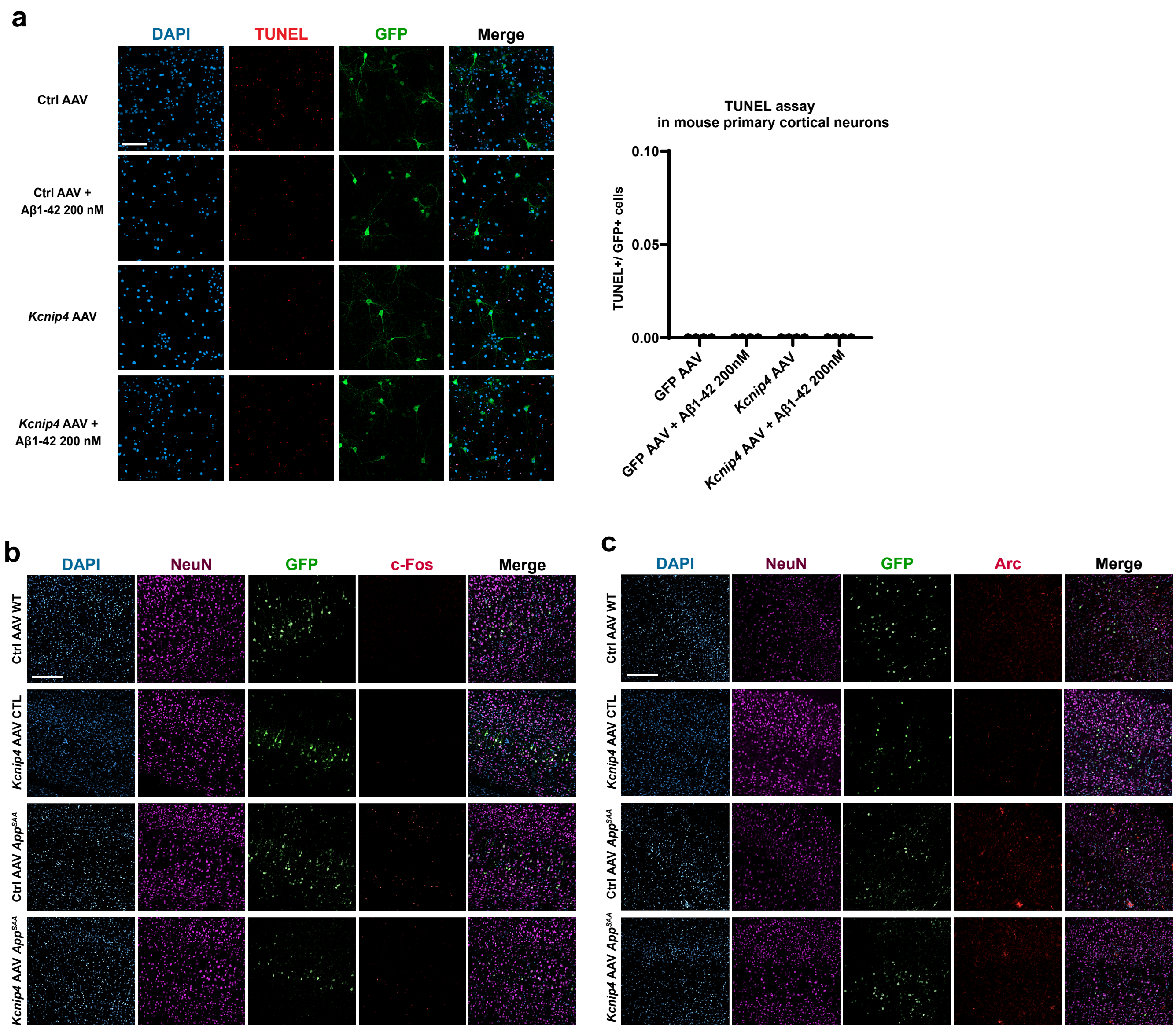

Supplementary Fig. 11. AAV-mediated delivery of *Kcnip4* in excitatory neurons

a, Representative images of TUNEL assay on primary mouse neurons at DIV14 after co-transduction with *Kcnip4* or control AAVs and jRGECO1a, with or without Aβ1-42 oligomer treatment, and quantification of TUNEL+ nuclei in transduced GFP+ neurons for each condition (four wells per condition, three fields per well). **b**,**c**, Representative low-magnification confocal images of SSC co-stained with c-Fos (**b**) or Arc (**c**) along with DAPI, NeuN, and GFP, used for c-Fos and Arc quantification in App^{SAA} and WT mice treated with *Kcnip4* AAV or control AAV. Scale bars: 100 µm (a); 200 µm (b,c). Source data are provided as a Source Data file.
