# Supplementary material for: Molecular signatures of resilience to Alzheimer’s disease in neocortical layer 4 neurons
Source: Nat Commun. 2026 Jan 31;17:2223. doi: 10.1038/s41467-026-68920-4 (PMC12963381; doi:10.1038/s41467-026-68920-4)
Supplement: Supplementary file 2 — Description of Additional Supplementary Files [file 41467_2026_68920_MOESM2_ESM.pdf]

Description of Additional Supplementary Files

Supplementary Data 1. Brain tissue samples used for snRNA-seq and Xenium.

Supplementary Data 2. snRNA-seq quality control (QC) metrics, including the DropletUtils thresholds for removing empty droplets, the number of doublets detected by DoubletFinder, and the total number of nuclei retained after QC (removal of nuclei with low gene counts, high mitochondrial content, and doublets) for each sample.

Supplementary Data 3. Gene markers for excitatory neurons (Ex 1-18), interneurons (In 1-19), and glial cell states, and gene sets (7-10 cluster-defining genes) for each cell subtype.

Supplementary Data 4. Numbers of nuclei within each neuronal cluster for each donor across regions and disease stages.

Supplementary Data 5. scCODA proportion analysis for BA9 and BA17.

Supplementary Data 6. Generalized Linear Mixed Model (GLMM) analysis of neuronal proportions for BA9 and BA17.

Supplementary Data 7. 'High-confidence' DE genes in excitatory neuron clusters across brain regions and disease stages.

Supplementary Data 8. Gene ontology and pathway enrichment analysis of 'high-confidence' DE genes using Metascape.

Supplementary Data 9. 'High-confidence' DE genes in clusters representing either vulnerable (Ex2; L2/3 IT) or resistant (Ex5; L4 IT) neuronal subtypes in BA9 and BA17 during early and late disease stages.

Supplementary Data 10. Co-expression modules identified through hdWGCNA for each excitatory cluster in BA9 and BA17.
