# Supplementary material for: Molecular signatures of resilience to Alzheimer’s disease in neocortical layer 4 neurons
Source: Nat Commun. 2026 Jan 31;17:2223. doi: 10.1038/s41467-026-68920-4 (PMC12963381; doi:10.1038/s41467-026-68920-4)
Supplement: Supplementary file 13 — Reporting Summary [file 41467_2026_68920_MOESM13_ESM.pdf]

nature portfolio

Corresponding author(s): Inma Cobos

Last updated by author(s): Dec 30, 2025

Reporting Summary

Nature Portfolio wishes to improve the reproducibility of the work that we publish. This form provides structure for consistency and transparency in reporting. For further information on Nature Portfolio policies, see our Editorial Policies and the Editorial Policy Checklist.

Statistics

For all statistical analyses, confirm that the following items are present in the figure legend, table legend, main text, or Methods section.

n/a	Confirmed	
☐	☒	The exact sample size (*n*) for each experimental group/condition, given as a discrete number and unit of measurement
☐	☒	A statement on whether measurements were taken from distinct samples or whether the same sample was measured repeatedly
☐	☒	The statistical test(s) used AND whether they are one- or two-sided<br>*Only common tests should be described solely by name; describe more complex techniques in the Methods section.*
☐	☒	A description of all covariates tested
☐	☒	A description of any assumptions or corrections, such as tests of normality and adjustment for multiple comparisons
☐	☒	A full description of the statistical parameters including central tendency (e.g. means) or other basic estimates (e.g. regression coefficient) AND variation (e.g. standard deviation) or associated estimates of uncertainty (e.g. confidence intervals)
☐	☒	For null hypothesis testing, the test statistic (e.g. *F*, *t*, *r*) with confidence intervals, effect sizes, degrees of freedom and *P* value noted<br>*Give P values as exact values whenever suitable.*
☒	☐	For Bayesian analysis, information on the choice of priors and Markov chain Monte Carlo settings
☐	☒	For hierarchical and complex designs, identification of the appropriate level for tests and full reporting of outcomes
☐	☒	Estimates of effect sizes (e.g. Cohen's *d*, Pearson's *r*), indicating how they were calculated

Our web collection on statistics for biologists contains articles on many of the points above.

Software and code

Policy information about availability of computer code

Data collection	No data collection software was used
Data analysis	kb python (0.26.0), DoubletFinder (4.2),python(3.9), scanpy(1.9.8), matplotlib(3.8.3), numpy(1.25.2), seaborn(0.13.0), scvitools(1.0.0) -includes scANVI and stereoscope-, R 4.2.0, lme4, MAST(1.32), pydeseq (0.3.2), hdwgcna(0.2.26), anndata (0.8.0), scikit-learn,sccODA 0.1.9, glmmTMB (1.1.11), Xenium ranger (3.1),spatialID (1.0.4),spatialdata (0.2.3), spatialdata_io (0.1.6), FIJI, ImageJ, Version 2.00-rc-69/1.52i (RRID: SCR_002285), CellProfiler (RRID:SCR_007358), GraphPad Prism 10 (RRID:SCR_002798). The scripts and the pretrained models are available at GitHub: https://github.com/AkilaRanjith/Molecular-Signatures-of-Resilience-to-Alzheimer-s-Disease-in-Neocortical-Layer-4-Neurons.git

For manuscripts utilizing custom algorithms or software that are central to the research but not yet described in published literature, software must be made available to editors and reviewers. We strongly encourage code deposition in a community repository (e.g. GitHub). See the Nature Portfolio guidelines for submitting code & software for further information.

Data

Policy information about availability of data

All manuscripts must include a data availability statement. This statement should provide the following information, where applicable:

- Accession codes, unique identifiers, or web links for publicly available datasets
- A description of any restrictions on data availability
- For clinical datasets or third party data, please ensure that the statement adheres to our policy

The raw snRNA-seq data, associated metadata, and processed digital expression matrices have been deposited at the NCBI's Gene Expression Omnibus with accession number GSE263468. Eight of 243 samples were included in previous studies (GSE129308 and GSE181715).
The snRNA-seqdatasets are publicly available for interactive viewing and exploration on the cellxgene platform at https://cellxgene.cziscience.com/collections/0d35c0fd-ef0b-4b70-bce6-645a4660e5fa
Xenium spatial transcriptomics data, associated metadata, and corresponding morphology files are available on Zenodo at https://zenodo.org/records/16703438

Research involving human participants, their data, or biological material

Policy information about studies with human participants or human data. See also policy information about sex, gender (identity/presentation), and sexual orientation and race, ethnicity and racism.

Reporting on sex and gender	We report the sex of each participant as detailed in Supplementary Table 1. Participants' sex was considered in analysis as a covariate.
Reporting on race, ethnicity, or other socially relevant groupings	We report the race of each participant when available in Supplementary Table 1
Population characteristics	The tissue samples were collected from three regions: the prefrontal cortex (BA9), precuneus (BA7), and primary visual cortex (BA17), encompassing all stages of disease progression. A total of 46 donors contributed to the study (42 for BA9, 15 for BA7, and 24 for BA17). The stages of disease progression were categorized into three groups: low pathology (18 donors; 6 females, 12 males), intermediate pathology (10 donors; 7 females, 3 males), and high pathology (18 donors; 12 females, 6 males). The criteria for each group were based on the presence and distribution of tau aggregates, according to the Braak staging system, and of amyloid pathology, including diffuse and neuritic amyloid plaques. The density of neuritic amyloid plaques was semi-quantified using the CERAD (C) staging system. The low pathology group included cases with no tau or amyloid pathology, with low AD neuropathologic change (ADNC), and cases of primary age-related tauopathy (PART). The PART cases in this study had a Braak stage I-III. The intermediate pathology group included cases with Braak stage III-IV and diffuse plaques or sparse (C1) neuritic plaques. The high pathology group included cases with Braak stage V-VI and moderate (C2) or abundant (C3) neuritic plaques. The mean age of the donors in the low, intermediate, and high pathology groups were 70.5, 81.9, and 82.4 years, respectively.
Recruitment	N/A
Ethics oversight	This work involves only post-mortem specimens and therefore received a regulatory determination of Not Human Subjects research.

Note that full information on the approval of the study protocol must also be provided in the manuscript.

Field-specific reporting

Please select the one below that is the best fit for your research. If you are not sure, read the appropriate sections before making your selection.

☒ Life sciences ☐ Behavioural & social sciences ☐ Ecological, evolutionary & environmental sciences

For a reference copy of the document with all sections, see nature.com/documents/nr-reporting-summary-flat.pdf

Life sciences study design

All studies must disclose on these points even when the disclosure is negative.

Sample size	Sample size was determined based on previous publications in the field
Data exclusions	Standard practices in snRNA-seq data analysis for excluding nuclei with low gene counts and doublets were used, no other data points were excluded.
Replication	Experimental findings were reliably reproduced across multiple independent biological replicates.
Randomization	For human donors, tissue was allocated to experimental groups based on Alzheimer's pathology staging and covariates were taken into account for the different quantifications as stated in specific methods sections, for mouse experiments, they were randomly allocated to control or experimental groups.

Blinding	Investigators were not blinded to experimental groups during data collection and analysis for snRNA-seq as the computational analysis required explicit metadata association (genotype/condition) to accurately map samples and perform differential expression analysis, although initial clustering was performed using unsupervised, data-driven algorithms. For mouse and human tissue histology, blinding was not feasible as presence of amyloid plaques significantly alters tissue architecture, rendering the genotype/condition visually apparent during microscopic imaging. To minimize potential observer bias, image analysis was performed using automated, custom CellProfiler pipelines that applied uniform parameters across all images without user intervention.

Reporting for specific materials, systems and methods

We require information from authors about some types of materials, experimental systems and methods used in many studies. Here, indicate whether each material, system or method listed is relevant to your study. If you are not sure if a list item applies to your research, read the appropriate section before selecting a response.

Materials & experimental systems

n/a	Involved in the study
☐	☒ Antibodies
☐	☐ Eukaryotic cell lines
☐	☐ Palaeontology and archaeology
☐	☒ Animals and other organisms
☒	☐ Clinical data
☒	☐ Dual use research of concern
☒	☐ Plants

Methods

n/a	Involved in the study
☒	☐ ChIP-seq
☒	☐ Flow cytometry
☒	☐ MRI-based neuroimaging

Antibodies

Antibodies used	Mouse anti-NeuN (1:1000, Millipore Sigma MAB377; RRID: AB_2298772)<br>Mouse anti-VGLUT2 (1:1000, Millipore Sigma MAB5504; RRID:AB_2187552)<br>Mouse anti-Kcnip4 antibody (1:1000, Proteintech 60133-1-Ig; RRID: AB_2130271)<br>Guinea pig anti-c-Fos (1:200, Synaptic Systems 226 308; RRID:AB_2905595)<br>Mouse anti-Arc (1:200, Synaptic Systems 156 111; RRID:AB_2631221)<br>Rabbit anti-GFP (1:1000, Invitrogen A11122; RRID:AB_221569)<br>Guinea pig anti-NeuN (1:200, Millipore Sigma ABN90P; RRID:AB_2341095)<br>Rabbit anti-EYA4 (1:50, ThermoFisher Scientific PA552113; RRID: AB_2641178)<br>Rabbit anti-human amyloid beta (1:500, IBL 18584; RRID: AB_2341377)<br>Rabbit anti-GFAP (1:2000, Dako Z0334; RRID: AB_10013382)<br>Rabbit anti-Iba1 (1:500, FujiFilm 019-19741; RRID: AB_839504)<br><br>Alexa Fluor 647 goat anti-guinea pig (1:200, Invitrogen A21450; RRID: AB_2535867)<br>Alexa Fluor 488 goat anti-rabbit (1:200, Invitrogen A11070; RRID: AB_142134)<br>Alexa Fluor 546 goat anti-mouse (1:200, Invitrogen A11018; RRID: AB_1500742)<br>Goat anti-mouse HRP (1:1,000, Invitrogen G-21040; AB_2536527)<br>Biotinylated goat anti-rabbit IgG (1:200, Vector BA-1000-1.5; AB_2313606)
Validation	All antibodies used were sourced from commercial vendors and were selected because they had previously been validated for use on human or mouse tissue in immunohistochemistry applications. We accounted for potential differences between lots and tissue fixation conditions by comparing the reactivity of the antibodies at three concentrations, and by including positive and negative controls.

Eukaryotic cell lines

Policy information about cell lines and Sex and Gender in Research

Cell line source(s)	*State the source of each cell line used and the sex of all primary cell lines and cells derived from human participants or vertebrate models.*
Authentication	*Describe the authentication procedures for each cell line used OR declare that none of the cell lines used were authenticated.*
Mycoplasma contamination	*Confirm that all cell lines tested negative for mycoplasma contamination OR describe the results of the testing for mycoplasma contamination OR declare that the cell lines were not tested for mycoplasma contamination.*
Commonly misidentified lines (See ICLAC register)	*Name any commonly misidentified cell lines used in the study and provide a rationale for their use.*

Palaeontology and Archaeology

Specimen provenance	*Provide provenance information for specimens and describe permits that were obtained for the work (including the name of the issuing authority, the date of issue, and any identifying information). Permits should encompass collection and, where applicable, export.*
Specimen deposition	*Indicate where the specimens have been deposited to permit free access by other researchers.*
Dating methods	*If new dates are provided, describe how they were obtained (e.g. collection, storage, sample pretreatment and measurement), where they were obtained (i.e. lab name), the calibration program and the protocol for quality assurance OR state that no new dates are provided.*

☐ Tick this box to confirm that the raw and calibrated dates are available in the paper or in Supplementary Information.

Ethics oversight	*Identify the organization(s) that approved or provided guidance on the study protocol, OR state that no ethical approval or guidance was required and explain why not.*

Note that full information on the approval of the study protocol must also be provided in the manuscript.

Animals and other research organisms

Policy information about studies involving animals; ARRIVE guidelines recommended for reporting animal research, and Sex and Gender in Research

Laboratory animals	AppSAA/SAA; B6.Cg-Apptm1.1Dnli/J (RRID: IMSR_JAX:034711); wild-type C57BL/6J (RRID:IMSR_JAX:000664), 12 month old. The facility was maintained under a standard 12-h light/12-h dark cycle, with a controlled ambient temperature of 22°C ± 2°C and relative humidity of 50% ± 10%. Mice had ad libitum access to water and standard chow.
Wild animals	Not involved
Reporting on sex	Due to the small sample size, we used only male mice for in vivo experiments in this study
Field-collected samples	Not involved
Ethics oversight	Protocol approved by Stanford's Administrative Panel on Laboratory Animal Care (APLAC)

Note that full information on the approval of the study protocol must also be provided in the manuscript.

Plants

Seed stocks	*Report on the source of all seed stocks or other plant material used. If applicable, state the seed stock centre and catalogue number. If plant specimens were collected from the field, describe the collection location, date and sampling procedures.*
Novel plant genotypes	*Describe the methods by which all novel plant genotypes were produced. This includes those generated by transgenic approaches, gene editing, chemical/radiation-based mutagenesis and hybridization. For transgenic lines, describe the transformation method, the number of independent lines analyzed and the generation upon which experiments were performed. For gene-edited lines, describe the editor used, the endogenous sequence targeted for editing, the targeting guide RNA sequence (if applicable) and how the editor was applied.*
Authentication	*Describe any authentication procedures for each seed stock used or novel genotype generated. Describe any experiments used to assess the effect of a mutation and, where applicable, how potential secondary effects (e.g. second site T-DNA insertions, mosiacism, off-target gene editing) were examined.*
